# Graphene Nanocomposite Membranes: Fabrication and Water Treatment Applications

**DOI:** 10.3390/membranes13020145

**Published:** 2023-01-22

**Authors:** Gorkem Memisoglu, Raghavan Chinnambedu Murugesan, Joseba Zubia, Aleksey G. Rozhin

**Affiliations:** 1 Department of Communications Engineering, Escuela de Ingeniería de Bilbao, University of the Basque Country (UPV/EHU), E-48013 Bilbao, Spain; 2 Department of Electronics Technology, Istiklal University, Kahramanmaras 46300, Türkiye; 3 Aston Institute of Photonic Technologies, Aston University, Birmingham B4 7ET, UK

**Keywords:** graphene nanocomposite membrane filter, carbon nanotubes, photocatalytic membrane, hybrid function membrane, membrane fabrication, water treatment

## Abstract

Graphene, a two-dimensional hexagonal honeycomb carbon structure, is widely used in membrane technologies thanks to its unique optical, electrical, mechanical, thermal, chemical and photoelectric properties. The light weight, mechanical strength, anti-bacterial effect, and pollution-adsorption properties of graphene membranes are valuable in water treatment studies. Incorporation of nanoparticles like carbon nanotubes (CNTs) and metal oxide into the graphene filtering nanocomposite membrane structure can provide an improved photocatalysis process in a water treatment system. With the rapid development of graphene nanocomposites and graphene nanocomposite membrane-based acoustically supported filtering systems, including CNTs and visible-light active metal oxide photocatalyst, it is necessary to develop the researches of sustainable and environmentally friendly applications that can lead to new and groundbreaking water treatment systems. In this review, characteristic properties of graphene and graphene nanocomposites are examined, various methods for the synthesis and dispersion processes of graphene, CNTs, metal oxide and polymer nanocomposites and membrane fabrication and characterization techniques are discussed in details with using literature reports and our laboratory experimental results. Recent membrane developments in water treatment applications and graphene-based membranes are reviewed, and the current challenges and future prospects of membrane technology are discussed.

## 1. Introduction

The environment we live in is basically composed of water, soil and air, and sustainable life can be maintained as long as this environment can be kept away from pollutants. In this context, one of the factors that can directly affect vitality and sustainability is water pollution [1,2,3,4,5,6]. Water is of vital importance for the survival of living things, and water pollution can easily occur with issues such as the discharge of wastewater containing harmful components into basins without adequate treatment. Fresh water resources on earth are limited, and it is extremely important to use water without wasting it, as well to support environmentally friendly water treatment applications [7,8,9,10]. Failure to ensure water sustainability can create many consequences such as drought-desertification (shortage of fresh water), poor quality water (polluted water), decrease in forestation (decrease in oxygen ratio in the world), decrease in agriculture (growth of unhealthy products), or increase in chronic diseases (due to a shortage of fresh water) [1,2,3,4,5,6,7,8,9].

In order to prevent these problems, it is important to use water treatment applications and to control the quality of natural, drinking, or artificial water sources and basins [11,12,13,14,15,16,17,18,19,20,21,22,23,24,25,26,27,28,29,30,31,32,33,34,35,36,37,38,39,40,41,42,43,44].

In water treatment systems, membranes are frequently used as a quality control medium combined with the filtering function. They are technical barriers of selectively permeable phases that allow water to pass through but prevent the passage of unwanted substances in water treatment [8,11,12,13,14,15,16,17,18,30,31,32,33,34,35,36,37,38,39,40,41,42,43,44,45,46,47]. New approaches and research are being carried out on water treatment in an environmentally friendly way using less energy, fewer chemicals, and fewer artificial light sources. In particular, the applicability of membrane technologies, which can provide excellent separation efficiency, modular structure, and low chemical sludge output, has been proven to help in water treatment [11,12,13,14,15,16,17,18,19,20,21,22,23,24,25,26,27,28,29,30,31,32,33,34,35,36,37,38,39,40,41,42,43,44,45,46].

Membranes are technical barriers that work like cell walls in the living body, filtering out particles like viruses or salts from water [11,12,21,22,23,24,25,26,27,42]. They can keep the filtered substances on their surface after filtering. In general, membranes with a porous and permeable structure are used in systems such as filtration-based water quality control, drinking or waste (domestic or industrial) water treatment, gas separation, dispersed solid (such as macromolecules or microorganisms) separation from water, air quality control, desalination, blood or urine dialysis in the environmental monitoring or biomedical field, so far as they meet sustainability criteria [11,12,13,14,15,16,17,18,19,20,21,22,23,24,25,26,27,28,29,30,31,32,33,34,35,36,41,42,43,44,48]. Membranes that form a barrier according to the type of contaminant offer advantages such as high effluent quality, less use of chemicals, low volume, and small footprint [11,12,13,14,15,16,17,18,19,20,21,22,23,24,25,26,27,28,29,35,36,37,38]. General contaminant removal methods are surface adsorption, biodegradation, membrane filtering, and photocatalytic degradation. Membrane filtering and photocatalysis processes in a water treatment system have different mechanisms with their own specific dynamics. Photocatalysis can provide microbial disinfection, prevent rapid fouling, and pore clogging of the membrane, while filtering processes like nanofiltration, ultrafiltration, reverse osmosis, or forward osmosis can lead to membrane fouling that can also be affected by the membrane pore properties like pore morphology and pore size or hydrophobicity [29,47,49,50].

In existing industrial membrane-based water treatment systems using polymer membranes made of materials such as cellulose acetate, polyamide, polyacrylonitrile, polypropylene, polyvinylidene fluoride (PVDF), polyether sulfone or polycarbonate (PC), filtration operates under high pressure (energy-dependency, driving forces like powerful filtration pumps) with periodic maintenance (demand for membrane cleaning, membrane replacements, and maintenance due to the rapid membrane fouling that increases the cost) and pretreatment process requirements, whilst the photocatalysis using chemical catalyzer materials is generally activated under ultraviolet light [9,27,30,51]. Such requirements can increase the total costs of the water treatment process [8,33]. So, although membrane technology has been used as a sustainable solution to water treatment technology, it still has limits. For membrane technologies to become a sustainable and cost-effective solutions, it is important to develop innovative membrane materials, nanocomposites and processes in photo-catalysis and membrane filtration [8,33].

**Figure 1:**
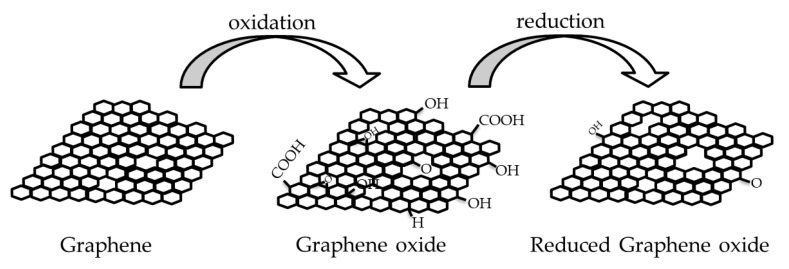
Forms of graphene, graphene oxide and reduced graphene oxide.

Graphene stands out with its high-performance and cost-effective production and processing methods, as well as being a green and sustainable material which cannot exist alone in nature as a single layer. The raw material of graphene is the graphite crystal that can be found in coal beds or metamorphic rocks like schist and gneiss. Until the 2004 discovery, many researchers tried to obtain graphene via physical and chemical methods [47,52]. In 2004, Novoselov and Geim exfoliated graphite and obtained graphene, a 2-dimensional, semimetal material with a hexagonal honeycomb carbon lattice structure, which has unique mechanical strength, thermal conductivity, and optical, electrical, chemical and photoelectric properties [47,52]. Figure 1 shows the chemical structures of a graphene layer, a graphene oxide which is the oxide form of graphene and a reduced graphene oxide.

Graphene-based membranes are one of the most important functional membrane materials and of great interest in water treatment applications, thanks to its excellent lattice structure anti-fouling and antimicrobial activities, and its contamination resistances [11,12,13,14,15,16,17,18,19,20,21,22,23,24,25,26,28,29,32,36,53,54,55,56,57]. As an example of the antimicrobial effect of graphene, a bacterial cell can be damaged and inactivated by the sharp edges of graphene [55], or bacterial cell walls can gain an inhibitory effect by the reduced graphene oxide [54]. Hu et al. investigated the antibacterial activity of graphene’s oxide form. They stated that the graphene sheets have sharp edges, which can cause membrane stress (membrane stress is the primary cause of cell death), disrupt membrane integrity of microbes (such as bacteria), and cause ribonucleic acid leakage, which can lead to the antibacterial activity of graphene [55]. Musico et al., investigated membrane filters with and without graphene, and they found that antibacterial properties were improved in the membranes with the graphene material [29]. Kumar et al. investigated the antibacterial activity of graphene’s reduced oxide form. They found that graphene exerted an inhibitory effect against bacteria (gram-positive bacteria and gram-negative bacteria) [54].

Graphene can be attached, coated or sealed onto a nano- or micro-porous polymer substrates like PVDF, nylon, PC or mixed cellulose ester with pore sizes between a few ten nanometers to micrometers to increase the mechanical durability of the membrane structure. Additionally, the most preferred graphene-based composite material combination in water treatment membrane technology is graphene with polymers like polyamide, PVDF, polytetrafluoroethylene (PTFE), cellulose acetate or PC. The reason for this preference is the mechanical, chemical, thermal and optical durability of the polymer medium [15,23,25,28,36,46]. Graphene oxide [16,17,18,20,23,24,29,32,36,58] and reduced graphene [12,13,22,26,43,54,59] are the main derivatives of graphene, and they carry a significant share of graphene properties. Oxidation can reduce the graphene’s aggregation tendency, and oxide form of graphene has both hydrophobic (graphene portion) and hydrophilic (like –COOH carboxyl group, or –OH hydroxyl group) edges that are shown in Figure 1. The difference between the oxide and reduced oxide forms of graphene is mainly in their functional groups. In graphene oxide, there are oxygenated functional groups, while in reduced graphene oxide there are few oxygen-containing functional groups. According to the dispersibility properties in aqueous media, graphene oxide shows high dispersibility, while reduced graphene oxide dispersibility is low. Although the reduced graphene oxide has similar characteristics to graphene, it is inferior in quality to graphene in terms of the presence of structural defects. Graphene oxide can be prepared by modified Hummers method, while its reduction can create reduced graphene oxide by low-cost thermal, chemical, or electrochemical methods [60]. Thanks to its amphiphilic nature, graphene oxide can bind to water-insoluble particles (by hydrophobic interaction, π-π stacking, or non-covalent bonding) [61]. This binding ability to insoluble particles, such as impurities, makes graphene an effective material for water treatment applications.

It is known from the literature that by nanoparticle doping into membranes, permeability, high temperature stability, separation performance, and selectivity of the membrane can be improved [45,62,63]. Therefore, nanoparticle doped graphene nanocomposite membranes have interest for us. Nanoparticles such as carbon nanotubes (CNTs) or metal oxide nanoparticles’ doping to the graphene can modify the surface morphology and improve the contaminant selectivity and water flux or contaminant removal [23,24,25,29,36,40,45]. Therefore, graphene:CNTs nanocomposites as water treatment membranes are promising for the ultrafiltration and fouling detection applications [15,23,43,44,45].

Both graphene and CNTs are forms of carbon materials. Like graphene, CNTs, which are tubes made of graphene layer(s) in metallic or semiconductor cylinders [23,27,30,59,64,65], show antimicrobial mechanism that may vary depending on physical and chemical effects and bacterial oxidation due to their electronic structures [56].

**Figure 2:**
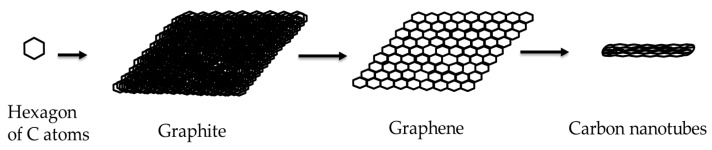
Forms of carbon materials; 3-D graphite, 2-D graphene and 1-D nanotube.

Figure 2 shows a hexagon structure of carbon, graphite crystal, graphene layer, and CNTs.

Characteristic properties of graphene and CNT membrane materials are summarized in Table 1. Beside graphene, CNTs also have exceptional, mechanical, thermal, electrical, electronic, and optical properties [56]. Due to their unique physical properties, CNTs are widely explored in many applications like materials science (additive materials, composites), photovoltaics, telecommunication, sensors, and bio-medical applications [66,67,68].

Graphene nanocomposites with CNTs can exhibit better antifouling property and pore structures than the pristine graphene membranes. Therefore, using CNTs in the structure of graphene composite is important [15,23,43,44,45]. Basically, CNTs are classified into two different classes based on the number of rolled-up graphene sheets, namely single walled CNTs (SWCNTs) and multiwalled CNTs (MWCNTs) [56,67]. The basic requirement for the synthesis of CNTs are a carbon source, catalyst, and adequate energy.

The utilization of transition metal catalyst leads to formation of SWCNTs, whilst the absence of catalyst results in the formation of MWCNTs. Three common methods, namely, electric arc discharge, laser ablation, and chemical vapor deposition (CVD), are generally used to synthesize CNTs, and are briefly described in the CNTs synthesis section. The SWCNTs and MWCNTs can be differentiated using transmission electron microscope (TEM) and Raman spectroscopy studies. SWCNTs exhibits unique chirality-dependent photoluminescence properties in the near infrared (NIR) regions, which give this class of material great potential for photoluminescence imaging and Raman/photoacoustic imaging. Moreover, the strong nonlinear optical absorption of SWCNTs makes them highly promising as a saturable absorber in high-power laser generation. MWCNTs lack optical properties due to their defects; however, they have been widely employed in many applications, such as field emission devices, radio frequency interference (RFI) shields, electrochemical capacitor or reinforcing additive materials [66,67,68]. Generally, pristine CNTs have difficulties in being dispersed in water or other common solvents due to their hydrophobic nature as well as Van-der-Walls attraction [22,24]. Therefore, different functionalization, using non-covalent and covalent approaches, have been developed to enhance the dispersibility/solubility of CNTs. For instance, in order to disperse the hydrophobic CNTs in water, functional groups are formed on their surfaces by using acid and dispersion of CNTs in water can be facilitated [22,24]. The noncovalent functionalization involves physisorption of surfactants, such as sodium dodecyl benzene sulfonate (SDBS) or sodium dodecyl sulfate (SDS) on the sidewall of the SWCNTs leads exfoliation of CNTs with enhanced dispersibility. The non-covalent approach is beneficial in terms of retaining intrinsic properties. However, desorption due to aging leads to unstable dispersion of CNTs, using covalent functionalization of carboxylic acid via surface oxidation using strong acids (sulfuric acid (H_2_SO_4_)/nitric acid (HNO_3_)), which leads to stable dispersion with high dispersibility or solubility. However, the disruption of *sp*^2^ lattice leads to enhanced defects and a complete loss of intrinsic properties [56,66,67,68].

Metal oxides like titanium dioxide (TiO_2_) or zinc oxide (ZnO) photocatalytic materials are invaluable in water treatment applications. Metal oxide semiconductors, such as ZnO and TiO_2_, have been extensively used because they are abundant, environmentally friendly, and have high electron mobility [21,22,32,34,59,64,74,75,76]. Nanocomposites with Graphene:CNTs:metal oxide show better charge carrier lifetime, mobility, antimicrobial activity, and contamination resistance [59,64,74,77,78,79], which are important parameters for the photocatalytic processes in applications like visible light active filtration, dye degradation, and fouling detection. Graphene nanocomposite membranes including CNTs and metal oxides are of great interest in photocatalysis and filtration membrane water treatment applications, thanks to their antimicrobial activities and contaminant resistances. The combination of graphene, CNTs, and metal oxide semiconductors is expected to improve the mobility of photo-generated electron hole pairs, and thereby enhance the charge carrier’s lifetime, and presents a potential nanocomposite structure [59,64,74,77,78,79] that is important for the photocatalysis process in water treatment. Beside this, external supports like acoustic signals can improve the membrane performance and lifespan in terms of protecting the membrane from contamination and help remove contamination in water treatment [33,80,81,82,83,84,85]. Therefore, the production of biocompatible, self-cleaning, sustainable and antibacterial membranes made of graphene, CNTs, and visible light active, metal oxide based, acoustically supported photocatalytic filtration membranes for use in water treatment could be one of the most important issues to be investigated.

The aim of this study is to: review the existing graphene and graphene nanocomposites for water treatment systems in terms of material and membrane properties, as well as synthesis and characterization methods; review current problem analysis of membrane-based water treatment systems; examine solutions to increase the membrane lifespan and performance while decreasing the total cost; and to discuss the future expectations/prospects regarding these systems.

Material synthesis, graphene nanocomposite membrane preparation processes, and characterizations are given in Section 2 and Section 3. In Section 4, Graphene nanocomposite membrane applications in water treatment applications are summarized. Section 5 presents the current challenges and the future prospects of membrane technology. Conclusions are given in Section 6.

## 2. Preparation of Graphene Nanocomposite Membranes

### 2.1. Graphene

Graphene nanocomposites stand out with their green and sustainable material composition, as well as being suitable for high-performance and cost-effective production and processing methods. There are various methods to prepare graphene membrane.

**Figure 3:**
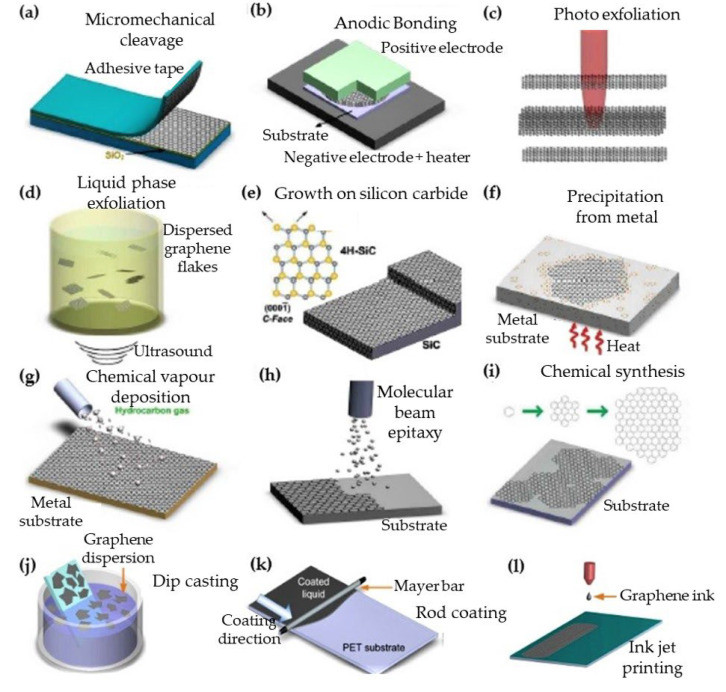
Graphene synthesis methods; (**a**) mechanical exfoliation, (**b**) anodic bonding, (**c**) photo exfoliation, (**d**) liquid phase exfoliation, (**e**) growth on silicon carbide, (**f**) precipitation from metal, (**g**) chemical vapor deposition, (**h**) molecular beam epitaxy, (**i**) chemical synthesis, (**j**) dip casting, (**k**) rod coating, and (**l**) ink-jet printing. Subfigures have been adapted/reproduced from reference [86] with permission from Elsevier.

Some of the methods used in the synthesis of graphene are: CVD (Figure 3g); chemical exfoliation (liquid phase exfoliation that can be obtained in a device like sonication-machine or shear mixer) (Figure 3d); physical exfoliation (mechanical exfoliation using sticky tape) of graphite, photo-exfoliation, precipitation, chemical synthesis, spin coating of reduced graphene oxide etc. (Figure 3a); anodic bonding (Figure 3b), photo-exfoliation (Figure 3c); graphene growth on silicon carbide (SiC) (Figure 3e); graphene precipitation from metal (Figure 3f); molecular beam epitaxy (Figure 3h); chemical synthesis (Figure 3i); dip-coating (Figure 3j); rod coating (Figure 3k); and ink-jet printing (Figure 3l) [86,87,88,89,90,91,92,93]. In these methods, materials like graphite, carbon, and graphene-ink can be used as the source material of the resulting graphene [86]. In Figure 3a–i, general graphene synthesis methods are shown, and Figure 3j–l show graphene preparation by graphene-ink source.

Some graphene synthesis methods are described in detail below.

CVD is suitable for mass production. Graphene can be grown on a heated catalytic metal foil substrate (like nickel or cupper) via a chemical reaction of gas-phase precursors (from gaseous carbon sources) in the CVD method [87,88,89,90] at high temperatures. An illustration of CVD is shown in Figure 3g. The basic steps of this method are as follows: the gaseous carbon source (reactant gas) is transported into the reactor, and first adheres to the heated catalytic metal foil surface by adsorption. After this, the graphene layer is formed on the metal surface by catalytic decomposition, cooling, and reorganizing of the surface carbon atoms to graphene. Many parameters such as the temperature, atmospheric pressure level, cooling rate, concentration of the carbon source, and solubility of carbon have impact on the graphene quality, such as the number of layers, layer dimension, and surface roughness of the produced graphene film.

**Figure 4:**
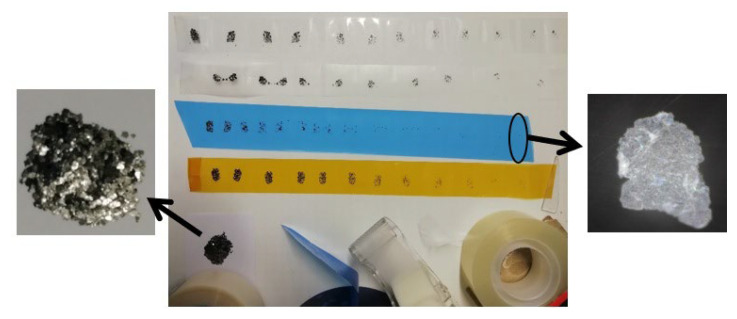
Natural graphite flakes’ mechanical (physical) exfoliation to graphene many-layers by adhesive tapes (picture and microscope image are taken in Universidad del Pais Vasco- Euskal Herriko Unibertsitatea—UPV/EHU laboratories, Bilbao, Spain).

In mechanical (physical) exfoliation (also called the tapping method or micromechanical cleavage) of graphite, the Van der Waals attraction forces between graphene layers are overcome and the layers are peeled off from the graphite, and graphene is obtained. Mechanical exfoliation of graphite to graphene layer(s) have been investigated in detail in the literature [47,52,86,94]. In 2004, physicists Novoselov and Geim at the University of Manchester achieved exfoliation of the graphite mineral by separating surface into layers by physical exfoliation, and obtained the first two-dimensional graphene plane with mechanical exfoliation method [52]. This green synthesis method is widely used in the manufacture of low-width graphene layers. An example of mechanical exfoliation process is shown in Figure 3a and Figure 4 where natural graphite flakes are used as the source material (from HQ-Graphene Inc., Groningen, The Netherlands) to be exfoliated by various adhesive tapes from Nitto Denko Inc., Osaka, Japan, Scotch-tape and 3M Inc., Saint Paul, MN, USA (Figure 4). After the graphite exfoliation processes, many layers of graphene are transferred onto the target (by the use of micromanipulators) from the adhesive tape (by wet-transfer where acetone: deionized water mixture was used as the interface medium). Graphene (on glass substrate) is visible under the optical microscope as shown in Figure 4. Another green coating method is dip casting (Figure 3j), which is useful for graphene composite membranes. A simple and green dip-coating method was used to prepare graphene oxide composite membranes by Lou et al. [95], and graphene on polymer by Khan et al. [17].

Liquid phase exfoliation, also called chemical exfoliation, is another method to prepare graphene layer(s), can be seen in Figure 3d. In the production of graphene from graphite by chemical exfoliation, the oxidation of graphite is ensured, and functional groups containing oxygen make graphite oxide which is hydrophilic and dispersible in water [96,97,98]. Chemical exfoliation of graphite is usually done in the presence of oxidants (to reduce the interaction between graphene layers).

In chemical exfoliation, chemicals like strong acids like sulfuric acid, potassium permanganate or sodium nitrate can be used as oxidants. Alkali metals are also used to exfoliate graphite and disperse the graphene in a liquid medium [94].

Electrochemical exfoliation is another method to prepare graphene. In electrochemical exfoliation, a working electrode (can be graphite rod or cylinder) and a counter electrode (can be graphite rod or cylinder) can be immersed in electrolyte solutions, with direct current (DC) applied to the electrodes (working and counter) for the exfoliation of graphite to graphene layer(s) [99].

Graphene can also be synthesized by surface precipitation of carbon in some transition metals as described in [87,91,100]. Furthermore, silicon desorption from silicon carbide single-crystal surfaces can give a multilayer graphene structure as described in [92,93]. Additionally, for the functionalization of graphene surface or in the reduction processes of graphene and its composites, low-cost and biocompatible materials can be used, and are called ‘green functionalization or ‘green reduction’ [75]. In green reduction processes, which is a kind of hydrothermal method, biological or biologically compatible sources like plant extracts can be used as the bio-reductants [75].

It is a challenging issue to be able to produce graphene using a sustainable and environmentally friendly solution, which can be applied at a large scale, with high quality and a low cost. Therefore, further development of these green production solutions, such as exfoliation or dip-coating in green graphene production, is very important. It should also be noted that graphene is sensitive to its substrate or framework-substrate material with which it is in contact. Thus, the material of the graphene substrate is a challenge. For practical and real time applications, it is difficult to find a suitable substrate that can be mounted under graphene. The most suitable substrate materials for graphene are SiO_2_ and silicon carbide [75,101].

### 2.2. CNTs and Graphene Nanocomposites with CNTs

**Figure 5:**
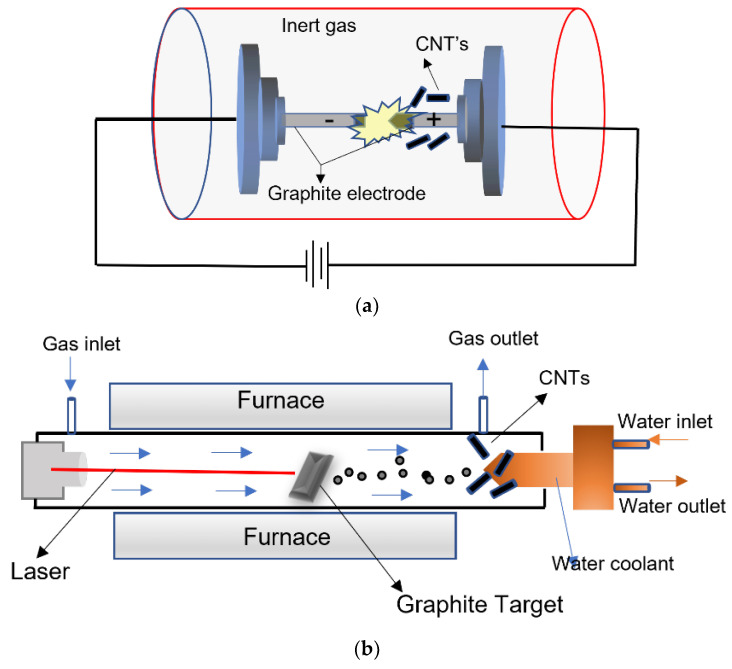

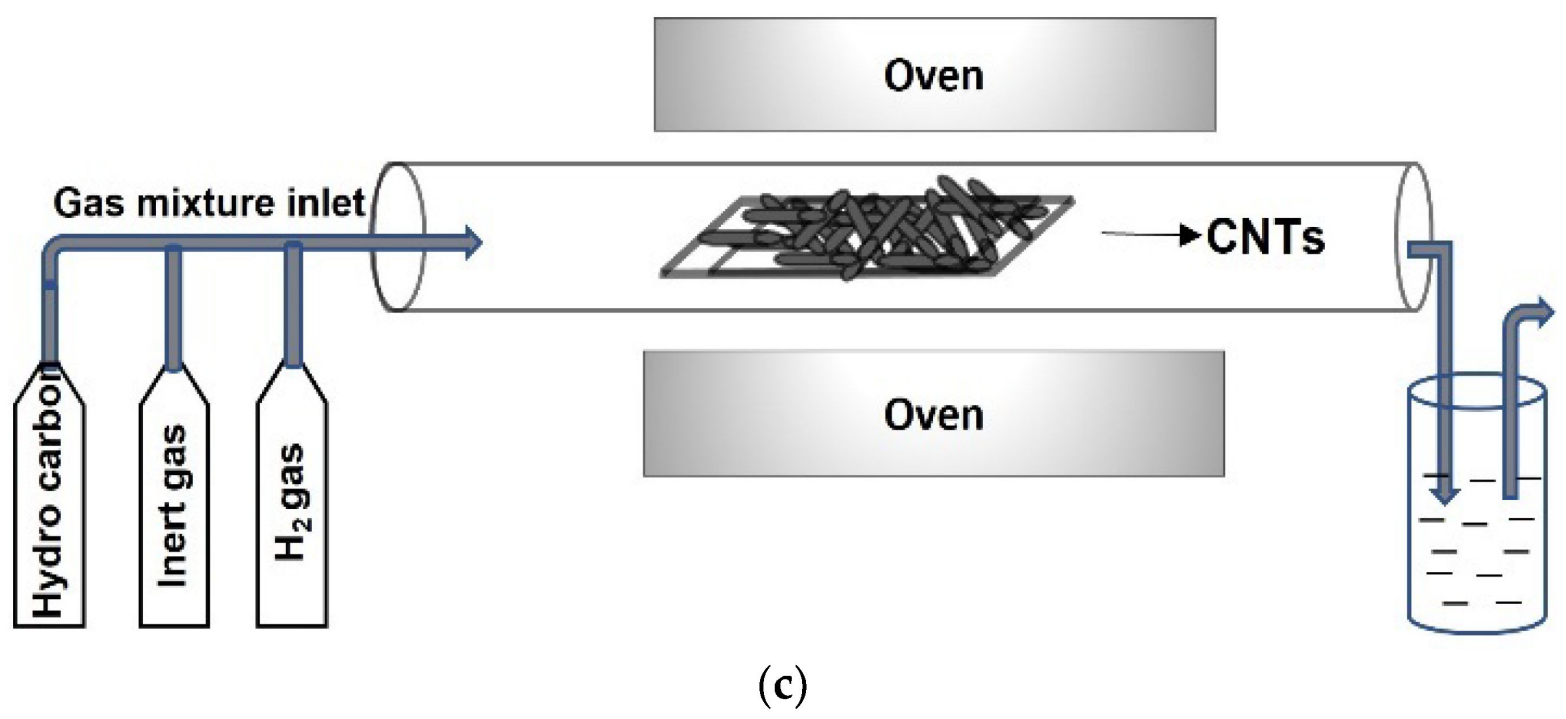
An illustration of CNT synthesis methods: (**a**) electric arc discharge, (**b**) laser ablation, and (**c**) chemical vapor deposition method.

CNTs can be functionalized and doped to the graphene structure. CNTs can be prepared by three standard methods, viz., arc discharge synthesis, laser ablation, and chemical vapor deposition [67,102]. These three methods are shown in Figure 5. Figure 5a shows the electric arc discharge method. Arc discharge, in which high power electric arc is generated between the high purity graphite electrodes (separated by the distance ~1–2 mm) in a chamber filled with an inert gas like helium or argon. The vaporization of graphite electrode by the passage of DC across the electrodes at a pressure high pressure (about 100 Torr) leads to the formation CNTs at the chamber wall or at the cathode. Both SWCNTs and MWCNTs can be synthesized by this method.

Doping of a metal catalyst—such as iron (Fe), cobalt (Co), nickel (Ni) or molybdenum (Mo)—on both electrodes leads to the formation of SWCNTs. By optimizing pressure, current, inert gas type, temperature, and system geometry, the quality and quantity of CNTs will be controlled.

Laser ablation is often preferred to synthesize high quality SWCNTs with an appropriate metal catalyst as used in the arc discharge method. In the laser ablation method (as shown in Figure 5b), the laser beam (mostly carbon dioxide (CO_2_) and neodymium-doped yttrium aluminum garnet; Nd:Y_3_Al_5_O_12_ (Nd-YAG) laser source) vaporizes a mixture of graphite and metal catalyst target in a closed horizontal tube under inert gas atmosphere at high temperature with controlled pressure. CNTs are deposited on a cooled surface of the hot furnace. The graphite target without catalyst leads to the formation of MWCNTs [67,102].

The CVD method (shown in Figure 5c) is considered to be a potential alternative to arc discharge and laser ablation to prepare CNTs due to its synthesis temperature that can be reduced to below 800 °C. Moreover, the CVD method provides a great degree of freedom in control of purity, yield, nanotube orientation, length, diameter, and density.

During CVD, the carbon feedstock, typically in the form of hydrocarbon gas was decomposed at a specified temperature into carbon gas deposited on transition metal catalysts such as Fe, Co, or Ni in the water cooling end of the quartz tube. Hydrocarbon sources such as methane (CH_4_), acetylene (C_2_H_2_), ethanol (C_2_H_5_OH), liquefied petroleum gas (LPG), and carbon monoxide (CO) are used for the synthesis of CNTs [67]. Catalytic CVD growth of CNTs can be achieved either thermal or plasma enhanced technique. Moreover, other techniques like water assisted-CVD, oxygen assisted CVD, hot filament-CVD (HFCVD), microwave plasma CVD (MPECVD), and radiofrequency CVD (RF-CVD) can be used for the synthesis of CNTs [67,102].

The combination of graphene and CNTs is promising for water treatment applications. The addition of CNTs to the graphene structure can improve the pore structure of membrane surfaces, hydrophilicity, and antifouling properties of the ultrafiltration and electrochemical filter membranes [15,23,43,44,45,103].

Synthesis of nanocomposites with graphene and CNTs can be completed using the following process: (i) graphene preparation by, e.g., mechanical exfoliation of graphite, (ii) functionalization of pristine CNTs, (iii) ultrasonication of graphene and CNTs in a liquid such as ethanol for dispersion.

### 2.3. Graphene Nanocomposites with CNTs and Metal Oxides

Graphene nanocomposites with CNTs can exhibit better antifouling property and pore structures than pristine graphene membranes. Therefore, graphene:CNTs nanocomposites as water treatment membranes are promising for the ultrafiltration and fouling detection applications. Furthermore, nanocomposites with graphene, CNTs, and a semiconducting metal oxide like TiO_2_ or ZnO have proven to exhibit high photocatalytic activity in dye degradation of contaminated water disinfection applications. The combination of graphene, CNTs, and metal oxide semiconductor is expected to improve the mobility of photo-generated electron hole pairs, and thereby enhance the charge carrier’s lifetime, and so presents a potential nanocomposite structure [59,64,74,77,78,79] that is important for the photocatalysis process in water treatment.

TiO_2_ metal oxide semiconductors have been extensively used because they are green, abundant, and have high electron mobility [59,64,76], which are important properties for green photocatalytic processes.

A synthesis example of a nanocomposite with graphene and metal oxide can be completed using the following process: (i) graphite mechanical exfoliation to graphene, (ii) treatment of graphene with chemicals (like H_2_SO_4_, KMnO_4_, H_2_O_2_, HCl), (iii) filtration and centrifugation, (iv) combining the resulting graphene suspension with metal oxide (like TiO_2_) and stirring, (v) heat treatment, (vi) washing with water, and (vii) freeze-drying treatment (to avoid from aggregate formation) as described in [104].

Synthesis of nanocomposites with CNTs and metal oxide usually involves a three-step preparation process: (i) functionalization of pristine CNTs, (ii) adsorption of metal ions/metal oxide on functionalized CNTs, and (iii) heat treatment. Typically, oxidation of CNTs with strong acids (H_2_SO_4_/HNO_3_) is used for the functionalization of carboxylic group (carboxylation).

The metal oxides can be directly attached to carboxylated CNTs through adhesion. Special linkers, such as amine terminated linker or mercapto-terminated linker, attach either acid treated CNTs or metal oxide nanoparticles for the combination. There are also various wet-chemical synthesis methods, such as hydrothermal technique, microwave-hydrothermal and facile sonochemical method. Some of the other methods on the synthesis of CNTs or graphene/metal oxide nanocomposites are the atomic layer deposition, CVD, sputtering, laser ablation, thermal evaporation, and electrochemical deposition as described in [59,64,79].

Synthesis of nanocomposites with graphene, CNTs, and metal oxides (like TiO_2_) can be completed using the following process: (i) dispersion by ultrasonication of graphene and CNTs in ethanol, (ii) titanium tetrachloride (TiCl_4_) addition to the suspension, (iii) magnetic stirring/refluxed/centrifugation, (iv) washing with water and ethanol, and (v) heat treatment as described in [74].

### 2.4. The Choice of Polymers for Composite Development

Polymers have reasonable prices, ease of preparation, diversity, resistance to certain chemicals or temperature values, and the ability to form interstitial structures. Thanks to the polymer membrane’s properties—such as selectivity, chemical and thermal resistances, and variety of polymer membrane structures—their usage as membranes can add value to water treatment applications [12,15,16,17,18,21,29,35,39,45,105,106].

Polymers like cellulose acetate, PC, polyamide or polyacrylonitrile are generally used in industrial membrane-based water treatment systems that require periodic membrane cleaning maintenance and membrane replacements. However, polymers like PVDF, PTFE, polyamide, poly-sulphone, poly-vinyl chloride (PVC), polyvinyl alcohol (PVA), nylon, PC, and mixed cellulose ester can be used in membranes as a mixture with carbon materials, or as perfect porous polymer-substrates for carbon-based layers to improve the mechanical durability of carbon-based membranes [12,15,17,18,20,21,29,35,39,45,46,106].

The most popular membrane materials in water treatment membrane applications are polymers like PTFE, PVDF, or PC [106,107]. However, the use of polymer and graphene in the same membrane composite structure provides many advantages in water purification. When graphene is added to the pure polymer membrane, the mechanical, thermal, and separation properties of the polymer membrane are improved. Thus, the polymer membrane will not be dissolved by the power solution during the filtration process [106]. Furthermore, hydrophilicity, structure of surface pores, antifouling property, and surface roughness of a membrane made of polymer such as PVDF can be improved when they are used with graphene and CNTs [22,23,24].

As a substrate or in a mixed form, polymer components in graphene-based composite membranes can act as supporting scaffolds and can increase the mechanical strength and water permeability of the composite membrane [12,15,17,18,21,29,39,45]. Moreover, thermo-resistant properties of polymers like polyvinyl carbazole (PVK) can improve the dispersion of graphene and graphene oxide. For instance, a graphene-based membrane filter with polymer medium on commercial cellulose membrane filter was prepared, and a perfect graphene and graphene oxide dispersion was achieved by a thermo-plastic polymer (PVK) addition [29]. Additionally, polymers like PVA can be used as an adhesive medium in graphene based nanocomposite membrane structures [45], and mucoadhesive polymer like polyacrylic acid (PAA) can be used with graphene in a membrane structure to improve the water permeability [21].

In polymer and graphene composite membrane preparations, polymer selection, method of polymer and graphene preparations, and the method of combining polymer and graphene, there are important challenges. Combining a polymer material and a graphene material can be completed using two methods. In the first method, graphene can be dispersed in a solvent, then the polymer and graphene solution can be homogenized by vigorous mechanical agitation. In the second method, the dispersion of graphene in the polymer solution can be achieved in an ultrasonicate device [106]. On the other hand, for a commercial polymer membrane and a graphene material, facile methods like filtering or spraying can be selected for the nanocomposite membrane fabrication. Regarding the commercial polymer membrane and a graphene material, one of the easiest ways to fabricate a composite membrane composed of polymer and graphene can be to filter the graphene solution through the commercial polymer membrane, as in [108]. In addition, graphene membranes can be prepared on a commercial polymer membrane by simple electrospray methods that allow the preparation of membranes with a large area and high hydrodynamic resistance [109].

### 2.5. Nanocomposite Membrane Fabrication Methods

Nanocomposite membrane fabrication methods can be differentiated by the type of material used in the membrane structure and by the target application area.

Some of the methods used to fabricate membranes are vacuum filtration [11,14,18,20,26,36,44,45], phase inversion [15], pumping, casting—hot press [20], rod-coating [40], dip-coating [17], and gravure printing [36].

**Figure 6:**
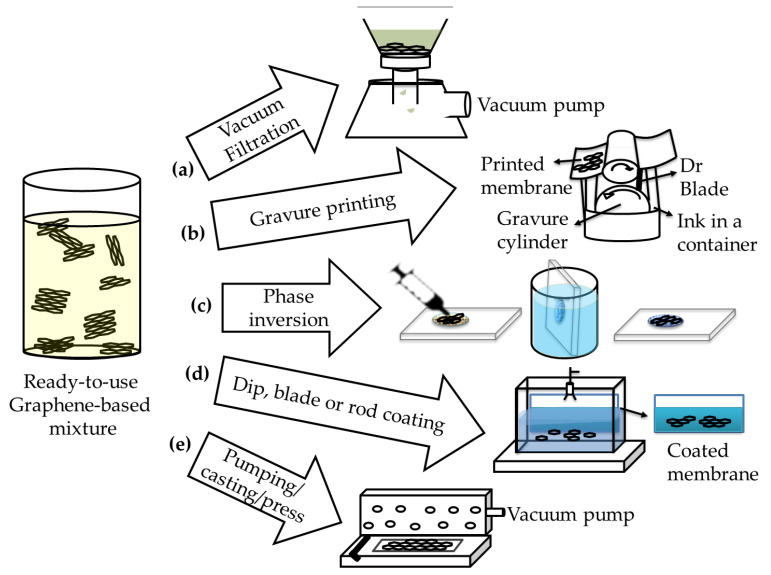
An illustration to the nanocomposite membrane preparation methods; (**a**) vacuum filtration, (**b**) gravure printing, (**c**) phase inversion, (**d**) dip-, blade- or rod-coating, and (**e**) pumping/casting/press with or without vacuum.

Figure 6 shows an illustration on the nanocomposite membrane preparation methods like vacuum filtration (Figure 6a), gravure printing (Figure 6b), phase inversion (Figure 6c), dip-, blade- or rod-coating (Figure 6d), and pumping/casting/press with or without vacuum (Figure 6e) [11,15,16,18,20,26,29,36,39,42,44,45]. Dip-coating, phase inversion, blade-coating, and rod-coating have minimal energy consumption, and are examples of green production methods of graphene composite membranes.

Graphene nanocomposites can be coated onto a polymer substrate by vacuum filtration, phase inversion, casting, and dip-coating methods [15,17,20,23,36,39,45]. For instance, Liu et al. used vacuum filtration method for the sonicated graphene-CNTs mixture to prepare graphene-based water purification membrane filter on PTFE polymer substrates [39]. In another study of Liu H. et al., freestanding ultrathin membranes made of reduced graphene oxide were prepared by vacuum filtration on a cellulose ester film substrate that was removed by acid treatment from the graphene layer at the end of the process to produce a freestanding graphene membrane [23]. In the study performed by Yuan et al., the phase inversion method was used where a polymer like PVDF was dissolved in a solvent like dimethyl sulfoxide or N-methyl pyrrolidone. In this method, the solvent used was precipitated from the coagulation bath. The coagulation bath was also miscible with the polymeric solvent to prepare graphene-CNT mixture [15].

In their 2020 study, Liu et al. prepared graphene-based membranes using the following method: after casting the graphene-based dispersion on a substrate like mixed cellulose ester, graphene-based mixture was sandwiched between the coated substrate and another mixed cellulose ester substrate. Finally, they used a hot press after putting the sandwiched membrane materials in a curable epoxy resin [20].

In gravure printing, small quantities of material are dropped on the printing plate where the liquid film and the underlying substrate must be well adhered. In this method, the film should not move on the underlying substrate to ensure uniformity and continuity of the film. A gravure cylinder (roller) helps to move the liquid, and a Dr Blade system was used to spread the small amount of liquid. After this, the substrate can be pressed by a rubber coated roller to transfer the liquid material from plate to the substrate to prepare to print large layers of membranes. Akbari et al. used a gravure printing machine to prepare a large area (e.g., 13 × 14 cm^2^) of graphene oxide nanofiltration membranes with thicknesses between 65 nm and 360 nm [36].

Dip-coating is another green method to prepare graphene-based membranes and was used by Khan et al. [17]. In their study, glass-fiber membrane substrate with graphene-based aqueous dispersion (with a soluble polymer or PTFE nanoparticles as a polymeric binder) were prepared by dip-coating. One of the other methods that can be scalable and facile is electro spaying, which was used by Chen et al. to prepare graphene oxide nano-filtering membranes [109].

### 2.6. Membrane Development

Graphene, CNTs, metal oxides like TiO_2_ and ZnO, and polymers like PVDF, PTFE, PVA, PC, and PVC, and in partial combinations together, are all good candidates to be used as membrane materials [8,9,11,12,13,14,15,16,17,18,19,20,21,22,23,24,25,26,27,28,29,30,31,32,33,34,35,36,37,38,39,40,41,42,43,44]. Graphene nanocomposite membranes with nanoparticles like CNTs exhibit high specific surface areas and photo catalytic activities which have been explored in water treatment applications [11,12,13,14,15,16,17,18,19,20,21,22,23,24,25,26,28,29,32,36,42,43,44,45,66,67,68]. Moreover, when the graphene material is doped with CNTs and metal oxide nanoparticles, the surface morphology can be improved, contaminant selectivity, water flux or contaminant removal properties can be improved [15,16,17,21,29,45].

Pristine graphene and graphene nanocomposites have been used in many studies to study photo catalytic activity for the water treatment including removal of dye and metal, water splitting, and antibacterial activity improvement [15,36,39,43,44,54,55,56,64,66,67,68,74]. When the antimicrobial activity of graphene was investigated, it was found that the graphene sheets’ sharp edges can be fatal for the bacteria by increasing the stress at the bacteria cell edges [55], and reduced graphene oxide can exert an inhibitory effect against bacteria [54]. Furthermore, antimicrobial mechanism of CNTs may vary depending on physical and chemical effects and bacterial oxidation due to electronic structure [56]. These findings regarding the antimicrobial activity of membrane materials are invaluable for graphene nanocomposite membrane-based water treatment systems.

Graphene membranes have been investigated in sustainable water treatment with low energy consumption, contaminant adsorption, antibacterial and anti-fouling activities, high organic solvent sorption, fast water transport, and filtering capabilities [11,12,13,14,15,16,17,18,19,20,21,22,23,24,25,26,27,28,29,30,31,32,33,34,35,36,37,38,39,40,41,42,43,44,45,46,47,50,52,53,54,59,64,65,74,105,108,110].

Table 2 summarizes the advances in graphene nanocomposite membranes including membrane preparation methods and key features.

Graphene filtering membranes prepared by a filtration assisted assembly method for water purification were investigated by Han et al. [11]. They achieved 21.8 L m^−2^ h^−1^ bar^−1^ pure water permeability, more than 99% organic dye retention and 20–60% ion salt retention with thin (22–53 nm thick) graphene nanofiltration membranes on microporous substrates. Joshi et al. prepared graphene oxide membranes by vacuum filtration, and investigated the filtration and salt separation in both experimentally and theoretically. They found that the fast salt separation can be thanks to the ion sponge effect (concentrated salt solutions’ rapid separation within graphene capillaries) [42].

Musico et al. used a graphene-based membrane filter with polymer medium on commercial cellulose membrane filter to investigate antibacterial properties. According to the results of their study, better dispersion of graphene and graphene oxide was found thanks to the polymer (polyvinyl carbazole), and the usage of the graphene-based membrane filters improved the antibacterial properties (greater bacteria cell damage with graphene) of membrane filters (for water and wastewater treatment) [29]. Seo et al. investigated the salt rejection, anti-fouling, and water flux properties of graphene on PTFE polymer membranes [53]. They achieved 99.9% salt rejection with Graphene/PTFE membrane, similar to the value with commercial PTFE membranes. They also observed that the graphene/PTFE membrane provides higher water vapor flow than the pristine PTFE membrane. Chen et al. prepared graphene oxide nano-filtering membranes by electro spraying, which is a more scalable and facile method than the traditional vacuum filtration [109].

Membranes made of graphene and CNTs are candidates for ultrafiltration and fouling detection, and can present improved properties on surface pore structures, surface roughness, hydrophilicity, and antifouling property [37,38,39].

Liu et al. prepared graphene-based electrochemical filter where CNTs were used as conductive binders in the membrane structure for water purification [39]. In their study, using vacuum filtration, graphene:CNT mixture was coated on to a commercial PTFE membrane which had 5 µm pore-sizes. Total thickness of the membrane was 15–20 µm, and the membranes had a 0.01 mol h^−1^ m^−2^ oxidation rate with 88% Tetracycline removal. Akbari et al. obtained 71 L m^−2^ h^−1^ bar^−1^ water permeability with high rejection (over 95%) for various dyes from the large-area graphene-based nanofiltration membranes that were prepared by gravure printing process [36]. Jha et al. prepared nano-filter membranes (by casting) with 95.21 μm thickness and 0.0267 µm pore-size, made of reduced graphene oxide to remove iron [12]. Prepared membranes had high metal contaminant (iron) rejection (95.77%). Gao et al. used graphene oxide with polymer materials in the membrane composition, and received 68.21 L m^−2^ h^−1^ bar^−1^ water permeability and more than 97% rejection of dye like methylene blue and congo red [21]. Yadav et al. prepared graphene based nanocomposite membrane where CNTs and PVA were used as a nano-spacer and as an adhesive, respectively [45]. They coated (by vacuum filtration) the nanocomposite on a hydrophilic mixed cellulose ester support (pore size 0.22 µm) to obtain a highly ordered laminated structure. They obtained 94.2% sodium sulphate rejection and 85.86% sodium chloride rejection with a high water permeate rate of 14.2–13.45 L m^−2^ h^−1^ at 5 bar pressure level. In addition, Liu H. et al. studied freestanding ultrathin membranes made of reduced graphene oxide to be used in water treatment [23]. The thickness range was between 20 nm and 200 nm in their study. Furthermore, recently, Liu T. et al. performed a theoretical study on a graphene-based membrane filter to discuss its mechanical properties and water permeation [20]. In their work, graphene strips are woven into the filter membrane. In this way, they suggested that the mechanical properties of the graphene-based membrane were increased significantly. Moreover, one of the newly developed composite membranes in water treatment is graphene oxide/MXene. Mxene is a 2D material that can be carbide or nitride based. With the addition of MXene to graphene oxide, nanocapillary channels were formed in the composite, and it was reported that the water permeability of the graphene oxide/MXene composite membrane increased [112].

Thanks to the graphene’s lightweight, hydrophobicity, anti-bacterial effect, and contaminant adsorption capabilities [15,64,74], the introduction of graphene into a photocatalysis system can enhance the photoactivity by forming synergic contact with metal oxide catalyst like TiO_2_ or ZnO [14,32,34,65,74,113]. The efficiency of the photocatalysis process—which is a photochemical reaction that occurs with the initiation of free radical mechanisms when the photon interacts with the material—varies according to the property of the used material. In metal oxide photocatalysts, TiO_2_ is the most commonly used biocompatible (used in most toothpastes and pharmaceuticals) metal oxide photocatalyst with its strong oxidation power, fouling resistance, antibacterial function, and low-cost, along with strong absorption in ultraviolet [9,27,113] that can be shifted to the longer wavelengths [9,27,114]. Jiang et al. and Fan et al. reported that graphene can improve the photocatalytic activity of TiO_2_ under visible light in bulk-form [77,79], or in a layer-by-layer form where TiO_2_ was coated on graphene layer [59,77,79].

While a high amount of energy is consumed by using a light source in ultraviolet-active photocatalysis application within the scope of disinfection with photocatalytic degradation, energy efficiency is provided by using daylight in visible light active photocatalysis. Therefore, the application of visible light-activated photocatalysis is more advantageous than ultraviolet-light activated photocatalysis which requires high-energy ultraviolet sources [9,27,113].

Chemical doping to TiO_2_ can shift its absorption from ultraviolet to the visible region [9,27,78,104,113,114,115]. For instance, gold, silver, or nitrogen doping to the TiO_2_ can shift its absorption region from ultraviolet to the visible [9,27,114], and can disinfect biological contaminants such as bacteria and viruses [27].

Visible light active, biocompatible, antifouling, self-cleaning coatings with TiO_2_ and graphene-based filtration membranes to be used in water treatment have been reported in literature [78,104,114,115]. Athanasekou et al. (2015) prepared graphene-based water treatment membranes with the nitrogen doped TiO_2_ [114]. While the most obvious advantage of Ag-doped TiO_2_ (Ag-TiO_2_) photocatalysts is that even at very low levels of visible light, they can be sufficient to inactivate biological contaminants/pollutants [9,27,113], nitrogen doped TiO_2_ can give more reliability to the water treatment system remaining sufficient without additional metal nanoparticles. An additional advantage is that nitrogen is abundant in our atmosphere [114].

Graphene, CNTs, and metal oxide composites show enhanced photocatalytic activity for the removal of dye and/or metal [15,59,64,74]. Vecitis et al. proposed an antimicrobial mechanism of CNTs which may vary depending on physical and chemical effects as well as bacterial oxidation due to the electronic structure [56]. CNTs or polymers can prevent restacking of graphene layers [59].

Bellamkonda et al. described the restacking prevention of graphene as CNTs create an additional electron transport layer, and the electron hole recombination rate reduces in the graphene:CNTs:metal oxide nanocomposite [74].

Graphene and CNTs together have high contaminant adsorption capacity with more porosity and large surface area to provide more contact between metal oxide and contaminant [59,74]. In the nanocomposites composed of graphene and CNTs have additional electron transport channels in blend and can accept photoexcited electrons, they can reduce recombination by improving electron-hole lifetime in photocatalysis to create reactive oxygen species [59,74]. Both CNTs and graphene are good electron acceptors for TiO_2_ metal oxide [74]. To investigate the photocatalytic activity for a dye degradation, metal oxide was used as a photocatalyst in the CNTs and graphene structure by Huang et al. [59].

Water permeability and membrane performance improvement have been investigated by the use of acoustic technology in water treatment systems [33,80,81,116,117]. It was reported that the acoustic stimuli can protect the membrane from fouling and help remove contamination [33,80,81,82,83,84,85].

In a patent invented by Gavalas (assignee is NASA), an acoustic actuator was used with microporous filtering membrane made of CNTs. The acoustic actuator, which allows the acoustic vibrations to spread over the membrane and contains a PVDF film layer in contact with the filtering membrane, was used to support the water flux through the membrane by stimulating it with acoustic vibrations [80].

In a study by Fung et al., acoustic technology was used to increase the performance of the system in terms of eliminating the cake-layer (or gel-layer) that increases the fouling on the membrane. In their study, membrane cake-layer was broken up and removed within 100 ms with acoustic support [81]. Therefore, it is necessary to contribute to the development of sustainable and environmentally friendly water treatment applications by evaluating filtering systems supported by acoustic technology, including membranes made of graphene-based nanocomposite materials, CNTs, and visible light activated metal oxide photocatalyst, which has been partially investigated in water treatment studies.

## 3. Characterizations and Characteristic Properties of Graphene Nanocomposites

### 3.1. Characterizations of Materials and Nanocomposites

Some good tools to characterize graphene layer(s) and CNTs are the scanning electron microscopy (SEM), atomic force microscope (AFM), transmission electron microscope (TEM), and Raman spectroscopy. Morphological analysis, aggregation state, defects such as adhesion defect in the CNTs wall, amorphous carbon amount in a CNT sample, diameter, and length and thickness can be determined by electron microscopy or atomic force microscopy.

**Figure 7:**
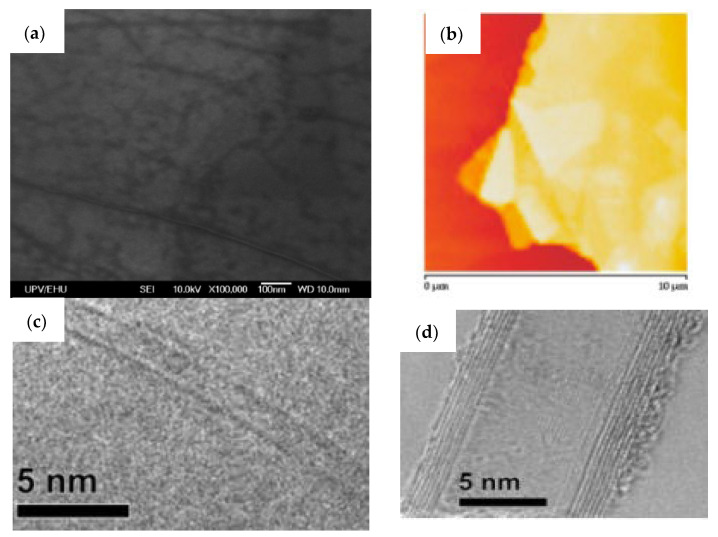
(**a**) Single-layer graphene’s SEM image (with 100 nm scale-bar), (**b**) many-layer graphene’s AFM image (with 10 µm scale-bar), and TEM images of (**c**) SWCNTs and (**d**) MWCNTs. Subfigures (**c**,**d**) have been adapted from [119] with permission from John Wiley and Sons. Subfigure (**b**) has been adapted from [118] with permission from MDPI. (SEM and AFM experiments in subfigure (**a**,**b**) are performed in Universidad del Pais Vasco/Euskal Herriko Unibertsitatea—UPV/EHU laboratories, Bilbao, Spain).

An example of an SEM image of single-layer graphene prepared by CVD on silicon/silicon dioxide substrate is shown in Figure 7a where the scale bar is 100 nm. Wrinkles and accumulations are presence in the single-layer graphene sample. Figure 7b shows an AFM image of a graphene many-layer sample with 10 µm scale-bar [118]. It can be observed that there are flakes of various widths in the many-layer sample.

TEM analysis can show the axial defects of a sample. Figure 7c,d show the TEM analysis of SWCNT and MWCNT samples, respectively. In TEM images of the samples, no defects are present in the SWCNT sample (in Figure 7c), whereas little defect concentration was found in the MWCNT sample (in Figure 7d) as described in [119].

**Figure 8:**
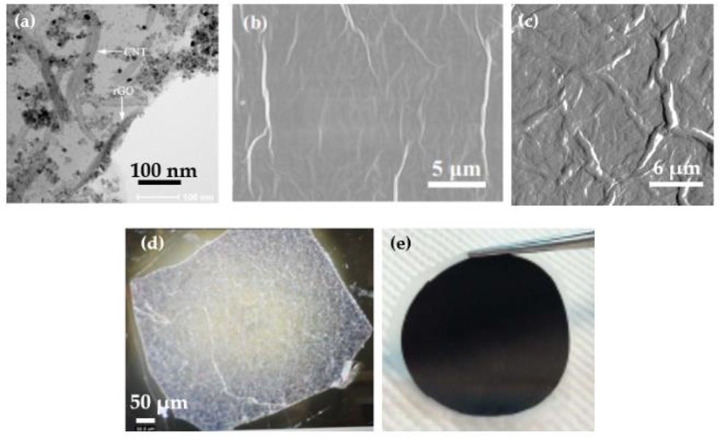
(**a**) TEM image of graphene oxide/CNTs/TiO_2_ layer [59], (**b**) SEM image of graphene oxide/CNTs membrane [37], (**c**,**d**) AFM and optical microscope images of graphene oxide membrane (performed in Universidad del Pais Vasco/Euskal Herriko Unibertsitatea—UPV/EHU laboratories, Bilbao, Spain), (**e**) a photograph of a graphene/CNTs membrane on PTFE [39]. Subfigures (**a**,**b**,**e**) have been adapted/reproduced from references [37,39,59] with permission from Elsevier, MDPI, and the Royal Society of Chemistry.

Sample images of the graphene nanocomposite membranes are given in Figure 8. A TEM image of graphene oxide/CNTs/TiO_2_ layer, a SEM image of graphene oxide/TiO_2_ membrane, an AFM image of graphene oxide layer, an optical microscope image of a graphene oxide membrane, and a photograph of a flexible graphene/CNTs membrane on PTFE are shown in Figure 8a–e, respectively.

**Figure 9:**
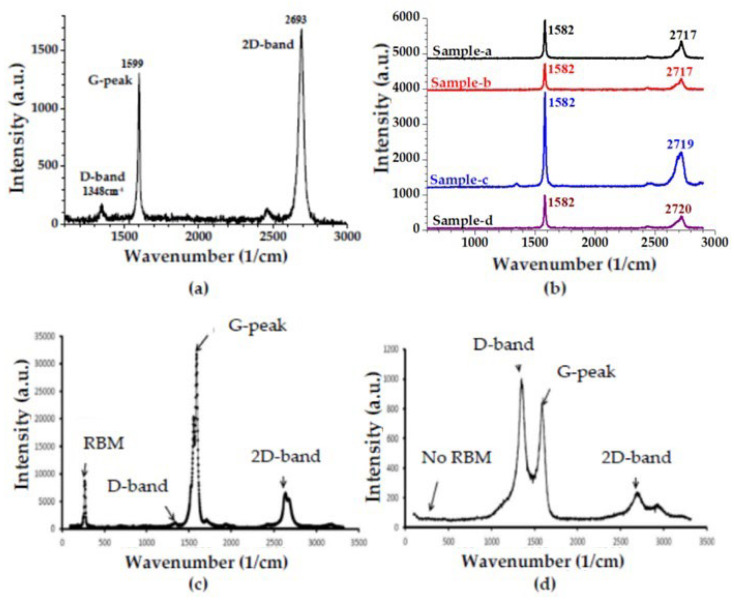
Raman spectrum of (**a**) single-layer graphene, (**b**) many-layer graphene, (**c**) SWCNTs and (**d**) MWCNTs. Subfigures (**c**,**d**) have been adapted from [123] with permission from Springer Nature. Subfigure (**b**) has been adapted from [118] with permission from MDPI. (Raman Spectroscopy experiments are performed in Universidad del Pais Vasco/Euskal Herriko Unibertsitatea—UPV/EHU laboratories, Bilbao, Spain. In Raman analysis in Figure (**a**), λ_exc_ = 532 nm).

Raman spectroscopy is a useful tool for investigating the characteristic peaks of a sample as well as obtaining information about the quality or contamination level of the sample. Homogeneity and quality of a graphene film on a substrate like SiO_2_, Si or Ni, can be evaluated by Raman spectroscopy. Furthermore, characteristic peaks of a graphene sample can be investigated by Raman spectroscopy. An example of a Raman spectrum in Figure 9a shows the characteristic G-peak, D-band, and 2D-band of graphene. In this spectrum, at 1348 cm^−1^ the D-band, at 1599 cm^−1^ the G-peak, and at 2693 cm^−1^ the 2D-band are visible for a single-layer graphene sample under 532 nm laser excitation source. Layer number, deformation level or disorder level of graphene sample can be interpreted by evaluating the intensities of the characteristic graphene peak and bands (like G, D, 2D) in Raman spectroscopy. Deformation and defect level of the graphene can be analyzed by investigating the D-band and G-peak intensity ratio (I_D_/I_G_). For instance, if the I_D_/I_G_ ratio is high, deformation and defect level of graphene can be low in a sample [118,120]. Figure 9b shows the graphene many-layers with various thicknesses without wavenumber shift in G-peak value (1582 cm^−1^) in the Raman spectrum of many-layer graphene samples (in this figure, the thinnest sample is sample-a, whilst the thickest sample is sample-d in Figure 9d). Calculated I_D_/I_G_ value of the samples are the highest (1.19) for the thinnest sample (sample-a in Figure 9b), and the lowest (0.04) for the thickest sample (sample-d in Figure 9b) which means that the deformation and defect level are low in the thinnest sample [118].

Raman spectroscopy can also help to determine the impurity level of a CNTs sample. For example, according to the characteristic Raman peak and band investigations of a CNTs sample, absence of the D-band can be attributed to a pure sample, or the radial breathing mode (RBM) band, which is sensitive to the diameter and can distinguish the CNTs as single walled or multi walled, can be clearly visible under the 785 nm red laser source excitation spectra that can show signals from semiconducting tubes (514 nm green laser shows the presence of metallic tubes), or quality of a CNTs sample can be determined by the D-band and G-peak intensity ratios [66,67,68,119,121,122,123].

Raman spectrum examples of SWCNT and MWCNT are shown in Figure 9c,d. Characteristic G-peak, D-band and 2D-bands of CNTs are present at 1575 cm^−1^, 1340 cm^−1^ and 2680 cm^−1^, respectively. The characteristic radial breathing modes (RBMs) in Raman spectra can only be observed for the SWCNTs [66,67,68]. The RBM, which represents the mode in which all carbon atoms in CNTs move simultaneously in the radial direction, is invisible in the MWCNT, is visible only in the SWCNT, and is seen at 142 cm^−1^, as can be seen in Figure 9c,d [122,123,124].

### 3.2. Characteristic Properties of Nanocomposite Membranes in Terms of Water Treatment

Sustainability, biocompatibility, and environmentally friendliness are important properties in nanocomposite membranes when using them in biological purposes like water treatment [62,63,125,126,127,128,129]. Nanocomposite membranes containing new filtration materials, nanofillers, polymers, or inorganic oxide materials can have thermal stability, high surface hydrophilicity, high water permeability, or high strength depending on the type of materials used in their structures [62,63,125,126,127,128,129].

In water treatment, the membrane filtration method is more advantageous than the traditional filtration methods as it consumes less energy and is therefore more economical, and membrane development studies are very important to solve the problems related to the need for clean water [15,42,126]. In addition to the membrane filtering process, the photocatalytic function combines filtration membranes, which are also called hybrid function membranes, and which perform better in matters such as organic contaminant (like dye) degradation/removal compared to pristine filtration. These have been investigated in the literature [50,78,115]. For an effective organic contaminant degradation, semiconductor material properties like band gap, surface area, particle size, porosity, and crystal structure are some important parameters [130]. In photocatalytic membranes, photocatalysis starts with the activation of a photocatalyst in the membrane structure via the light absorption. Light absorption, which can be altered by recombination of charge carriers generated by photogeneration, can cause a change in the number of photocatalyst light active sites and limit the charge carrier [131].

**Figure 10:**
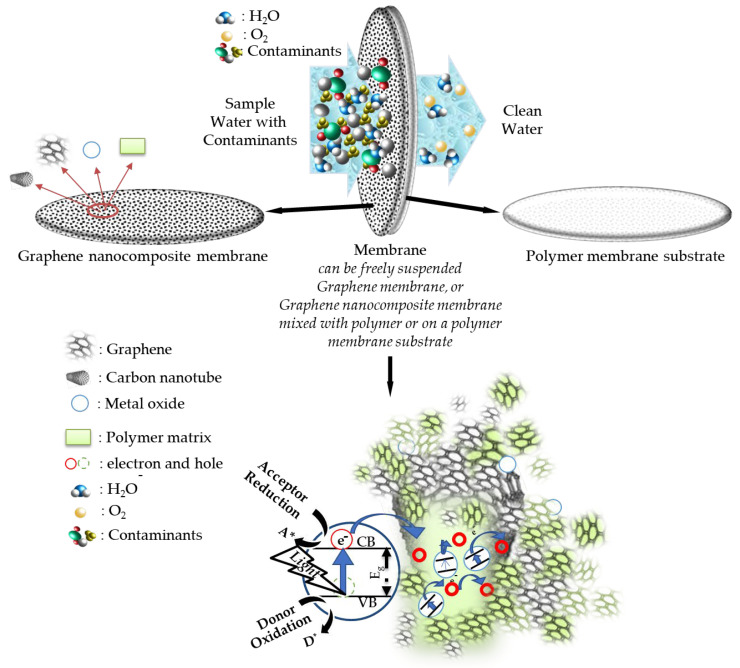
Schematic examples of a membrane structure, water permeation process, and a photocatalysis process.

After the bandgap energy (Eg) of the semiconductor is exceeded by the light absorption, through the redox reaction in the semiconductor, the electron is excited from the valence band (VB) to the conduction band (CB), forming electron hole pairs, and reactions of electron transfer happen (like radical formation), which finalizes with contaminant desorption to the compounds like mineral acids, and then transfers back to the inlet water sample. An example of such a process can be seen in Figure 10.

In studies on nanocomposite photocatalysis, thanks to providing additional electron transport channels in blend, accepting photoexcited electrons, and reducing recombination by improving electron-hole lifetime in photocatalysis to create reactive oxygen species, graphene and CNTs are good electron acceptors for the TiO_2_ photocatalyst [59,74]. Figure 10 depicts a general lab-scale membrane structure, water permeation process, and photocatalysis process illustrations.

Photocatalytic water treatment membranes can be made of functionalized composites like graphene, CNTs, metal oxides, and polymers. Functionalization is a chemical surface treatment and a basic technique that adds new properties/capabilities to the material. The use of functionalized membrane materials and additives in the membrane structure can improve membrane performance or reduce membrane fouling in terms of water permeability, selectivity, and high pollutant rejection [16,17,21,29]. Membrane material functionalization can be in the form of attachment of functional groups to the surface of the material layer. In the functionalization of the graphene layers, functional groups (like nanoparticles or polymers) can be attached to the surface of the graphene layer by covalent bonding [94,104].

Nanoparticle doping can improve some properties of membranes such as the permeability, stability (at high temperature), and separation performance, and the selectivity trade-off of the membrane can be eliminated by nanoparticle doping [45,62,63]. CNTs or metal oxide nanoparticles can be added to the graphene nanocomposite membrane structures to change the membrane’s morphology, and to modify the surface properties to improve the mechanical properties, water flux, and contaminant selectivity/removal through the membrane [23,24,25,29,36,40,45]. However, if the number of nanoparticles in the nanocomposite is not adjusted, the membrane functions like water flux may decreases. For example, Yadav et al. found that increasing CNT concentration in a graphene nanocomposite membrane decreases the membrane’s contaminant rejection rate and water flux [45]. On the other hand, one of the main problems with the nanoparticle: polymer type composite membrane is that nanoparticles form a fine dispersion around the polymer structures [127]. In addition to the composites, membranes made of pristine materials have also been investigated for their freestanding forms, such as graphene freestanding membranes [13,46,102]. Although the pristine graphene layers show high mechanical strength, freestanding graphene membranes can suffer from some structural problems such as stability, continuous strength, and application/process difficulties like applicability to large areas or separation in an aqueous medium [23,46,102].

In nanocomposite filtering membranes, pore-clogging is a crucial issue to be overcome. Clogging of the membrane pores increases both the rapid decline in membrane performance and the fouling tendency of the membranes [8,9,11,12,13,14,15,16,17,18,30,31,32,33,34,35,36,37,38,39,40,41,42,43,44,46,47,49,50]. Furthermore, membranes’ water permeability, undesired material rejection, flux recovery, long-term viability, reusability, and contaminant retention capabilities are important parameters for the water treatment applications.

Permeation can be analyzed by a dead-end stirred cell-filtration unit (in a chamber filled with nitrogen gas). The water permeability rate of graphene membrane can be increased with increasing applied pressure and flux. The water permeability (pure water flux) *M* of a filtration membrane can be defined as follows [36,44];

(1) $$M=\frac{V}{A\;\Delta P\;\Delta t}$$

where *V* is the permeated water volume, *A* (m^2^) is the membrane’s effective area, ΔP (bar) is the pressure difference and Δt (hour) is the permeate time. Permeability is in L m^−2^ h^−1^ bar^−1^.

The rejection (separation) percentage *R* of a membrane can be defined as [44];

(2) $$R=\left(1-\frac{C_{permeate}}{C_{feed}}\right)\times 100\%$$

where $C_{permeate}$ is the permeate solution concentration and $C_{feed}$ is the feed solution concentration. Furthermore, the membrane’s flux recovery is as follows [36,44];

(3) $$\mathrm{Flux}\;\mathrm{recovery}\;\left(\%\right)=\left(\frac{J_{w,i}}{J_{w,1}}\right)\times 100$$

where $J_{w,1}$ is the initial water flux before the first cycle and $J_{w,i}$ is the flux after cycle *i*.

## 4. Water Treatment Applications of Graphene Nanocomposite Membranes

Earth’s precious freshwater resource can be contaminated by agrochemicals, pharmaceutical derivatives, heavy metals, and endocrine disruptors being somehow mixed into the water resources [10,63,127,128,129]. The production of membrane materials that are resistant to agrochemicals, pharmaceutical derivatives, heavy metals, endocrine disruptors, heat, and oxidation are invaluable in membrane development. Graphene nanocomposite membranes have received great attention thanks to properties such as fouling resistance, antimicrobial activity, and improved membrane lifetime. Several nanometers thick layers made of graphene are of great interest because of graphene’s unique physical, chemical, optical, and electrooptical properties [12,13,14,15,16,18,21,29,32,37,40,41,42,44,45,47,52,64,74].

Ultrafiltration or micro filtration and forward osmosis or reverse osmosis techniques (using hollow or spiral shapes membranes) are the most used ones in current water treatment membrane technologies, which generally operate under high pressure and ultraviolet light [9,21,65].

Polymer-based membranes, which are traditionally used in commercial systems, facilitate the continuous operation of the membrane in water permeability processes. However, what really plays the role of selective permeability are graphene-based nanocomposite membrane components. Graphene nanocomposite membranes can be more useful for cost-effective water treatment, including water desalination, filtration, purification, dye or metal degradation in water ultrafiltration, purification, contamination detection or water separation applications [15,21,74]. Graphene membranes have better hydrophilicity, antifouling properties, pore structures, and surface roughness compared to the pristine polymer membranes. Furthermore, graphene membranes’ antibacterial activity, hydrophilicity, water flux, and fouling properties can be improved when they are used in a modified composite membrane structure with materials such CNTs, polymer, or metal oxides [15,74].

Modification and functionalization of membrane surface imparts new properties to the membrane, and can increase the utility of membranes in separation processes by improving the antifouling properties and stability of the membranes [17,18,30,35,37].

In addition to modification and functionalization, the use of sound waves in membrane-based water treatment systems for issues such as improving the water flow through the membrane, preventing contamination of the membrane, and impurity accumulation on the membrane surface, as well as prolonging its life, is an environmentally friendly application that does not require the use of any chemicals [26,82,116,132].

Sound waves in the 20–20 kHz frequency range (acoustic) and more than 20 kHz frequency (ultrasonic) can support water flow and filtering capabilities of filtering membranes [26,82,116,132,133,134]. Furthermore, ultrasonic frequency categories are the power ultrasonic frequency ranging from 20 kHz to 100 kHz, the high-frequency ultrasonic from 100 kHz to 1 MHz, and the diagnostic ultrasonic from 1 MHz to 500 MHz. In industrial cleaning processes like membrane surface cleaning or pollution prevention, 20 kHz to 500 kHz are often used. Thanks to the drag force acting on the membrane surface with acoustic or ultrasonic support, impurities on the membrane surface can be removed [116,133]. Acoustic or ultrasonic frequency sources include acoustic chips, piezoelectric chips, and ultrasonic horns [26,82,116,132,133].

**Figure 11:**
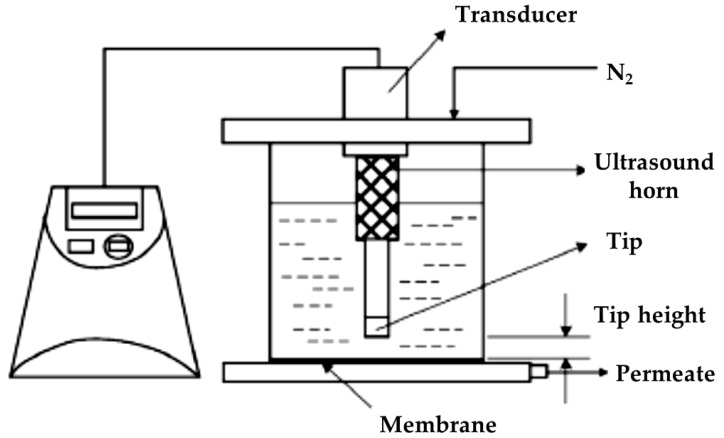
Experimental setup example for an ultrafiltration of suspended solutions with ultrasound. Figure has been adapted/reproduced from reference [116] with permission from Elsevier.

Figure 11 shows a set-up of an ultrafiltration membrane that is supported by ultrasound which was produced by a transducer whose immersed tip is not in contact with the membrane [116]. In the ultrasound-assisted membrane system, cavitation caused by ultrasound waves is stated as the main factor that facilitates the passage of liquid material through the membrane [116]. Furthermore, high ultrasound intensity helps to clean the membrane surface, keeping the membrane clean by pushing the contaminants away from the membrane surface [116,134]. However, it has been reported that in these applications, especially at ultra-high frequencies, bubble formation on the liquid and membrane surface may adversely affect filtration, and membrane damage due to vibration should be prevented [26,82,132].

Water pollution and pollutants—which are one of the today’s ongoing environmental problems, are likely to be encountered by the membrane in water treatment processes and are still being studied to remove them from water—can be classified as organic and inorganic. Organic pollutants in a water sample can be dyes or phenol derivatives, while inorganic pollutants can be inorganic salts or toxic heavy metals. Graphene nanocomposites can adsorb and remove both the organic and inorganic pollutants from water [135].

Membrane adsorption technology is an economical and rapid method to remove both organic and inorganic contaminants. However, it should also be noted that most of the reports in the literature are the results of experimental investigations of pollution structures with predefined properties in the laboratory environment. However, in the real world, water resources and water in the waste class can have pollution structures with very different components [135,136,137].

### Effects of Graphene Nanocomposite Membranes Produced by Green Methods on Water Treatment Applications

Environmentally friendly processes in water treatment applications have drawn attention. The most environmentally friendly water treatment applications are likely to be systems that do not contain harmful chemicals or consume high energy, and are combined with green materials, green production methods, and processes for a sustainable environment [74,75,138,139,140,141].

Green materials, green functionalization, green reductions, and green production methods mean that the material or process are environmentally friendly and sustainable. For instance, TiO_2_ photocatalyst is known as a green material [76], and mechanical exfoliation is a green production method for graphene without any need of energy or hazardous chemicals. The source material of graphene is green graphite crystal that can be found in nature. Furthermore, using green materials like plants in functionalization of graphene surface or reduction of a material like graphene oxide, or using visible light active nitrogen doped TiO_2_ photocatalyst in water treatment, are some examples of the biocompatible green processes [74,75]. Graphene nanocomposites are one of the green materials and have sustainable material compositions. They can be produced by green production and processing methods. Green membrane production method examples can include mechanical exfoliation, dip-coating, blade-coating, rod-coating, and phase inversion. The advantages of green membrane production methods are their low energy consumptions, easy and low-cost processes [141].

Green production method can refer to innovative system design, equipment and process methods. Reducing production cost, energy consumption, and equipment size and cost are examples of improvements for a greener production method. Membrane-based technologies are open to improvements in their green production methods [142].

Membranes, which are widely used in membrane-based water treatment technologies, are generally accepted as green water treatment technology [75,138,140]. However, with the widespread use of membranes prepared with green production methods in water treatment technologies, both the total production cost will decrease and sustainable development will be supported without harming nature and living things.

Today, instead of using pristine polymer membranes in water treatment, it is necessary to develop, produce by green production methods, and use nanostructured membrane materials which have higher efficiencies and energy savings than the polymer membranes [108]. According to the researches carried out to date, it can be seen that the promising technologies are the green technologies based on their advantages. Therefore, both the green materials and the green production method developments are crucial for the future of sustainable water treatment. However, more research needs to be done on this issue for green technologies to become established in water treatment.

## 5. Challenges and Future Prospects of Membrane Technology

The development of sustainable, environmentally friendly, cost-effective, high-performance, and long-lasting service life water treatment systems is critical to tackling global water quality challenges, and various studies have been undertaken to reduce environmental footprints and cost while increasing fouling resistance, antimicrobial activity, and membrane lifetime [8,9,12,14,15,16,17,18,19,20,21,22,23,24,25,26,27,28,29,30,31,32,33,34,35,36,37,38,39,40,41,42,43,44,45,46,47,50,52,54,55,56,59,64,65,74,105,110]. High cost and technical issues are the challenges of the current water treatment membrane technology [8,33]. In most of the water treatment systems using membranes, cost can be increased while using filtering processes like nanofiltration, ultrafiltration, reverse osmosis, and forward osmosis processes, and during photocatalysis processes there can be striking costs for materials (membrane, catalyst), energy requirements (for the pumps at high pressure-assisted ones, and ultraviolet source requirement for the photocatalysis), and periodic maintenance (demand for the membrane cleaning or membrane replacements) [8,33]. Therefore, there are still challenges to overcome for a sustainable and cost-effective membrane technology in water treatment technology.

A few of the ways to overcome these challenges include the development of photocatalysis systems and the use of innovative membrane materials, composites, and processes in filtration [8,33,64]. Photocatalysis processes with visible light active materials are reported to be low-cost and long-lasting water treatment structures [64]. Therefore, one of the most important issues to be investigated in water treatment may be filtration membranes based on graphene for the production of visible light active photocatalysis, biocompatibility, self-cleaning, and antibacterial effects. Both membrane replacement/maintenance and operation costs of the existing systems can be remedied by developing novel visible light active anti-fouling membranes with acoustic operation, leading to water flux support and extended service life [19,26,27,30,80,81,116].

High pressure usage as a driving force in membrane-based water filter system is another challenge that needs to be overcome.ost-effective water treatment systems are being studied by researchers, as are examples of a water treatment system that will not consume energy without also applying pressure, or a water treatment system that will consume less energy with low pressure applications [9,20,27,30,93].

Besides the cost issues, there are also technical challenges with water treatment, which can be explained as follows:

Synthesis of high-quality graphene is still under development and is considered a key task in most publications [48,143]. In addition, it is seen that such sustainable and high-performance materials are being developed with more emphasis on production with green production methods such as chemical exfoliation [143]. Furthermore, although the graphene production method, such as liquid phase exfoliation, also called electrochemical exfoliation, is an environmentally friendly method, there are still challenges associated with large area and high quality membrane production [141,143]. In addition, CVD has been used to prepare large area and high-quality membranes. However, CVD also has challenges such as energy consumption and gas relationship that may be harmful to health. It is not, therefore, green.

The issue of preventing the pore clogging of membranes after use is one of the challenges that needs to be solved in membrane technology. The challenge of membrane fouling (like biofouling, this means either microfouling or macrofouling) has been sought to be overcome by developing antifouling membranes. Membrane pores can be filled easily by bio-pollutants (biological microbes such bacteria (microfouling), or warm oyster (macrofouling) organisms) [34].

Rapid water filtration is a challenge in water treatment systems. Thin and freestanding membranes are desired for the rapid water filtration in water treatment systems, but as the membrane becomes thinner, many properties such as mechanical strength decrease [13,46]. Although graphene with its perfect mechanical strength in ultrathin layer structure can be used as a stand-alone membrane [13,46,118], the mechanical strength and lifetime of these freestanding membranes need to be improved to be used as filters in water treatment systems [13,46].

Another challenge is that the membrane holds or traps the organic or inorganic contaminants such as dye, paints, resins, metal particles, bacteria, viruses, or maggots, and prevents the rapid passage of clean water. The accumulation of all this pollution on the membrane surface can create problems. Filtering the contaminants by the membrane, breaking down the contaminant by photodegradation on the membrane surface, returning the decomposed substances to the inlet water, and preventing the adhesion of the contaminants to the membrane are the challenges that need to be overcome [13,34,46].

A material that is added to the membrane structure during the production phase of the membrane can be a biological compatibility challenge for the water treatment. For this reason, as long as the membrane structure is not damaged, the substances in its structure should not mix with the purified water and should be harmless. Although it is known that the materials in the structure of the membrane will not mix with the treated water as long as they do not break down or deteriorate, studies on biologically harmless materials are still ongoing [78,104,113,114]. For instance, according to the membrane filters research based on visible light activated graphene and nitrogen doped TiO_2_ [114], nitrogen doped structures provide more reliability to the water treatment systems since they do not contain additional metal particles such as silver nanoparticles.

Regarding these issues, antibacterial and self-cleaning materials and membranes have been investigated in literature [29,54,55,56]. Visible light active photocatalyst and antifouling membranes can provide extended service life without using catalyst chemicals in the inlet water [9,21,65,74]. Researches continues on visible light activated photocatalysis materials and coatings with graphene-based filtration membranes, which are also biocompatible, self-cleaning, antibacterial, and energy-saving thanks to the daylight usage [9,27,78,104,114].

In water treatment systems, there are also challenges related to the development and optimum use of acoustic technology support, which helps prolong system performance and membrane life by increasing membrane-to-membrane water flow and membrane filtration capabilities [26,82,116,132]. In prior art, acoustic pressure usage in water treatment has been mentioned in studies where the challenges like membrane damages due to the bubbles created at high frequencies were discussed [26,82,132].

Consequently, the research area of graphene nanocomposite structures, preparation, characterization and use in water treatment applications is still a hot topic [141]. Besides all these challenges and the options for overcoming them, the acoustic excitation at, e.g., the intersection of the underwater acoustic and human hearing frequency ranges support mechanisms still remain uncertain for the water treatment membranes made of graphene nanocomposites, and is open to further development in future studies.

## 6. Conclusions

Membranes clean water by filtering out dissolved salts, removing harmful organic and inorganic contaminants, and by improving the smell and taste. Membrane technology development is important as it determines the technological and economic efficiency of the operating processes in water treatment applications. Although membrane technology has been used as a sustainable solution to water treatment technology, there are still some challenges to overcome like using high energy consumption with strong vacuum pumps for water permeation processes, artificial light sources with photocatalyst chemicals for bio-disinfection processes, fast membrane fouling and short membrane lifetime, and inefficient and high-cost membrane structures. Therefore, it is essential to develop innovative membrane materials/composites and filtration processes to provide a sustainable and cost-effective solution. Furthermore, since the use of biocompatible, self-cleaning, antibacterial, and low-cost membranes in water treatment is one of the most important issues, it is necessary to contribute to the research and development of sustainable and environmentally friendly water treatment practices.

The most frequently used techniques in current membrane technologies are forward or reverse osmosis, and ultra or micro filtration techniques using hollow or spiral shapes to operate under high pressure and ultraviolet light. All these techniques require frequent membrane replacement and maintenance due to their rapid membrane fouling which is a significant cost source due to requiring powerful filtration pumps, high-energy ultraviolet-light sources, thermal support, and the use of catalyst chemicals to be released into the inlet water are some of these challenges. According to recent studies, sustainable and environmentally friendly water treatment applications can be further enhanced by the development of filtration systems powered by acoustic technology, including visible light active photocatalytic membranes made of graphene, CNTs, and a low-cost visible active metal oxide photocatalyst. Visible light active photocatalysis membranes can solve membrane fouling, and the cost issue can be solved by the using solar energy, a renewable energy source without needing high-energy ultraviolet-light sources and catalyst chemicals added to the treatment system, while supporting antimicrobial properties of the membrane. Thus, some of the key factors promoting the application of membranes are the improved performance by extended membrane service life with antifouling capability and acoustic support, and improved environmental and eco-friendly applications without additional catalyst chemicals to be released in water or thermal support.

In terms of innovation and the future of membrane technology, both membrane replacement/maintenance and operating costs of existing systems can be eliminated by developing visible light active membranes, made of graphene-based nanocomposites, supported by acoustic operation, which can provide longer service life is still open to development.

## Figures and Tables

**Table 1:** Characteristic properties of graphene and CNTs [67,69,70,71,72,73].

Key Features/ Advantages	Graphene			CNTs	
	Exfoliated Graphene	Graphene Oxide	Reduced Graphene Oxide	Single Wall	Multi Wall
Chemical structure	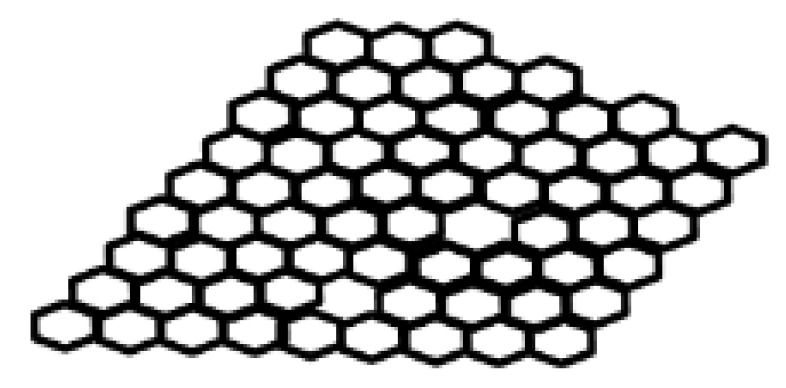	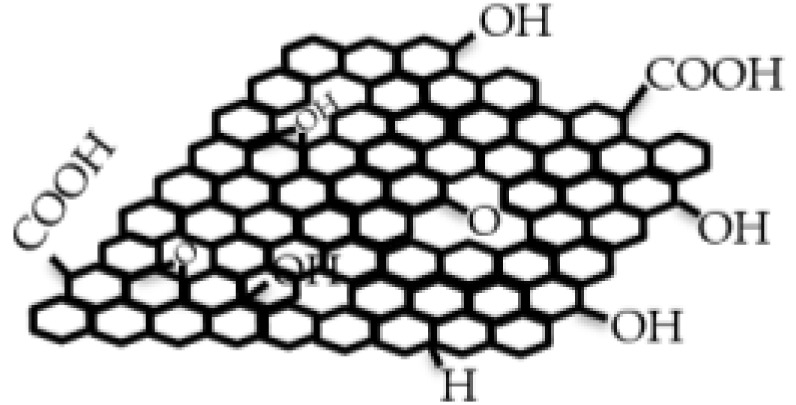	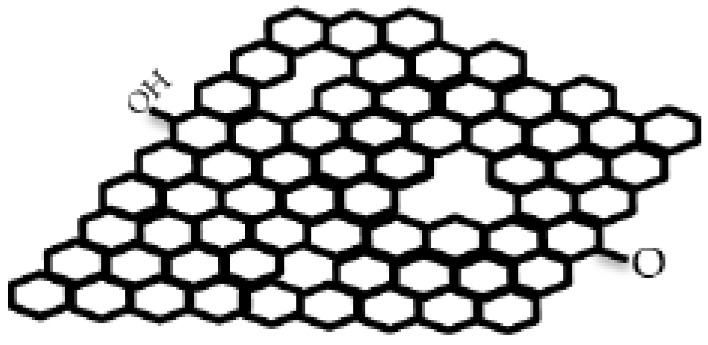	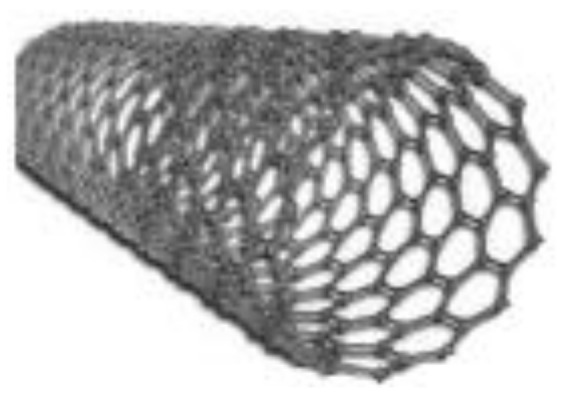	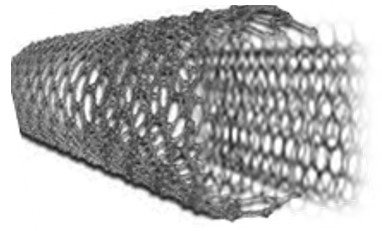
Elastic modulus (TPa)	~1	>1	>1	~1.4	0.3–1
Specific surface area (m^2^/g)	341–392	759 ± 198	669 ± 113	400–900	200–400
Thermal stability in air (°C)	600–800	600–800	600–800	600–800	600–800
Characterization	Easy	Easy	Easy	Easy	Difficult
Bulk or massive production	Relatively difficult	Easy	Easy	Difficult	Easy

**Table 2:** Advances in graphene nanocomposite membranes and properties.

Ref.	Membrane Composition (and Treatment Type)	Membrane Preparation Method/ Physical Properties	Key Features/Advantages
[11]	Graphene (nanofiltration)	Vacuum filtration	21.8 L m^−2^ h^−1^ bar^−1^ water permeability, high organic dyes and ion salts retention (>99%). Retention rate: 20–60%
[12]	Graphene + polymer (nanofiltration)	Casting Thickness: 95.21 μm Pore size: 0.0267 µm	High metal contaminant (iron) rejection (95.77%)
[14]	Graphene oxide + metal oxide (nanofiltration)	Heterogenous nucleation and diffusion-controlled growth process	225 L m^−2^ h^−1^ bar^−1^ water permeability, and up to 98% selectivity in the size-exclusion separation of methyl blue
[15]	Graphene oxide + CNTs + (PVDF; polyvinylidene fluoride) (ultrafiltration and fouling detection)	Phase inversion	High water flux of 125.6 L m^−2^ h^−1^ Improved surface pore structure and surface roughness, hydrophilicity, and antifouling property as compared with that of pristine PVDF membranes
[17]	Graphene + polymer on glass fiber (ultrafiltration)	Dip-coating (commercial glass fibre membrane was soaked in the mixture of Graphene (prepared by liquid phase exfoliation) and soluble polymer binder solution)	Improved selectivity (by ×10^3^ compared to the neat glass fibre membrane)
[18]	Graphene + polymer (purification)	Vacuum filtration	Ultrafast water permeability while remaining high rejections
[20]	Cellulose ester/ graphene oxide on cellulose ester support (filtration)	Pumping/casting and hot pressed	21.34 L m^−2^ h^−1^ bar^−1^ water permeability, with 96.08% salt rejection rate, 35.8% energy-saving in the membrane filtration process
[21]	Graphene + polymer (purification)	Vacuum filtration	68.21 L m^−2^ h^−1^ bar^−1^ water permeability, high rejection (over 97%) for dyes (like methylene blue, Congo red)
[23]	Reduced graphene oxide (purification)	Vacuum filtration Thickness: 0.02–0.200 μm, Diameter: 4 cm	Freestanding ultrathin graphene-based membranes
[29]	Graphene + polymer + graphene oxide on cellulose nitrate support (filtration)	Vacuum filtration Pore size: 8 µm	Better dispersion of graphene and graphene oxide (thanks to the polymer), greater bacteria cell damage.
[35]	CNTs/PTFE on poly-ethylene grid support (distillation)	Vacuum filtration Pore size: 0.2 μm	12 kg m^−2^ h^−1^ water permeability (flux rate) and 99.9% salt rejection
[36]	Graphene oxide on nylon substrate (nanofiltration)	Dr-Blade (5 × 5 cm^2^), Gravure printing (13 × 14 cm^2^), and Vacuum filtration Thickness range: 0.15 ± 15 µm Substrate pore size: 0.2 μm	71 L m^−2^ h^−1^ bar^−1^ water permeability, high rejection (over 95%) for various dyes
[37]	Graphene + CNTs	Electrophoretic deposition and chemical reduction	Improved water flux, high rejection (~94.0%)
[38]	Graphene + CNTs + graphene oxide	Vacuum filtration Thickness: 1.23 µm	52.7 L m^−2^ h^−1^ bar^−1^ water permeability, high rejection (over 98%) for dyes (such as methylene blue)
[39]	Graphene + CNT on polymer (PTFE; polytetrafluoroethylene) (purification)	Vacuum filtration Thickness: 15–20 µm Pore size: 5 µm	0.010 mol h^−1^ m^−2^ oxidation rate with 88% tetracycline removal
[40]	Graphene oxide (purification and molecular separation)	Rod-coating	60.0 kg m^−2^ h^−1^ water permeability and a high separation efficiency (~96.0%) for a sodium sulfate
[42]	Graphene oxide (filtration)	Vacuum filtration Thickness: 1 μm Pore size: 0.2 μm	0.2 L m^−2^ h^−1^ bar^−1^ water permeability
[44]	Graphene oxide (purification)	Vacuum filtration	10,000 L m^−2^ h^−1^ bar^−1^ water permeability, high rejection (~100%) for dyes (like methylene blue, rhodamine B)
[45]	Graphene + Polymer (PVA; polyvinyl alcohol)/ CNT on cellulose ester support (water treatment)	Vacuum filtration Pore size: 0.22 µm	Nanocomposite improved the separation performance (94.2% sodium sulphate and 85.86% sodium chloride rejections with high permeate rate (14.2–13.45 L m^−2^ h^−1^ at 5 bar))
[46]	Graphene oxide: bacterial cellulose (molecular separation)	Vacuum filtration	Freestanding graphene-based membranes
[53]	Graphene/PTFE (desalination)	Ambient-air CVD and wet-transfer	99.9% salt rejection, antifouling, long-term flux stable membranes
[57]	Graphene oxide/ silicon nitride/silicone (ionic sieving)	Thickness: 3 µm 200 × 200 nm^2^ membrane	~10^−4^ mol cm^−2^ h^−1^ ion permeation rate, 96% ion selectivity
[108]	Graphene oxide/ niobate nanosheet (nanofiltration)	Vacuum filtration 7.07 × 10^−4^ m^2^ membrane	20 L m^−2^ h^−1^ bar^−1^ water permeability
[109]	Graphene oxide/ nylon microfiltration membrane (nanofiltration)	Electro spraying 100 mm diameter membrane	11.13–20.23 L m^−2^ h^−1^ bar^−1^ water permeability, more than 98.88% organic dye rejection
[111]	Graphene oxide + silicon dioxide: PTFE	Layer by layer self-assembly, Dip-coating (commercial PTFE immersion/soaking in solution)	560.2 L m^−2^ h^−1^ water flux 50% fouling inhibition
[112]	Graphene oxide/ MXene on mixed cellulose ester	Vacuum filtration Thickness: 550 nm	71.9 L m^−2^ h^−1^ bar^−1^ water permeability High dye rejection (100%)

## Data Availability

The data presented in this study are available on request from the corresponding author.
